# Cost-Effectiveness Analysis of Capecitabine Plus Oxaliplatin Versus Gemcitabine Plus Oxaliplatin as First-Line Therapy for Advanced Biliary Tract Cancers

**DOI:** 10.3389/fphar.2022.871262

**Published:** 2022-07-22

**Authors:** Ruijia Chen, Yalan Zhang, Kongying Lin, Defu Huang, MaoJin You, Yanjin Lai, Jinye Wang, Yingying Hu, Na Li

**Affiliations:** ^1^ Department of Pharmacy, Mengchao Hepatobiliary Hospital of Fujian Medical University, Fuzhou, China; ^2^ Department of Pharmacy, The Second Affiliated Hospital of Fujian Medical University, Quanzhou, China; ^3^ Department of Hepatopancreatobiliary Surgery, Mengchao Hepatobiliary Hospital of Fujian Medical University, Fuzhou, China; ^4^ Department of Pharmacy, Mindong Hospital of Fujian Medical University, Ningde, China; ^5^ Department of Pharmacy, Fujian Medical University Union Hospital, Fuzhou, China; ^6^ The School of Pharmacy, Fujian Medical University, Fuzhou, China

**Keywords:** cost-effectiveness, advanced biliary tract cancers, XELOX, GEMOX, first-line treatment

## Abstract

**Background:** In the first-line treatment of biliary tract cancers (BTCs), XELOX (capecitabine plus oxaliplatin) showed comparable clinical efficacy and safety to gemcitabine and oxaliplatin (GEMOX), with fewer visits and better treatment management. Our study aims to investigate the cost-effectiveness of XELOX and GEMOX as the first-line therapy for BTCs from the perspective of the Chinese healthcare systems and to provide valuable suggestions for clinical decision-making.

**Methods:** A Markov model was developed using the phase 3 randomized clinical trial (ClinicalTrials.gov number, NCT01470443) to evaluate the cost-effectiveness of XELOX and GEMOX. Quality-adjusted life-years (QALYs) and incremental cost-effectiveness ratios (ICERs) were used as the primary outcomes of the model. Uncertainty was assessed using univariate and probabilistic sensitivity analysis.

**Results:** The QALYs for the XELOX and GEMOX groups were 0.66 and 0.54, respectively. In China, the total cost of XELOX treatment is US $12,275.51, which is lower than that of the GEMOX regimen. In addition, XELOX is more effective than GEMOX, making it the preferred regimen. A sensitivity analysis determined that XELOX therapy has a stable economic advantage in China.

**Conclusion:** Compared to GEMOX, XELOX is a more cost-effective treatment as a first-line treatment for advanced BTC from the perspective of the Chinese health service system.

## Introduction

Biliary tract cancers (BTCs), including cholangiocarcinoma (both intrahepatic and extrahepatic) and gallbladder cancer, are low-incidence cancers carrying a poor prognosis (Bergquist and von Seth, 2015; Gamboa and Maithel, 2020; Valle et al., 2021). BTCs account for approximately 3% of all gastrointestinal tumors and are predominantly adenocarcinomas (Doherty et al., 2017). Surgery is the main treatment for localized disease (Bridgewater et al., 2016), and most patients (>65%) are diagnosed with the unresectable disease. There is a high recurrence rate in the minority of patients who undergo potentially curative surgery (Valle et al., 2017). The 5-year survival rate for BTCs is approximately 5–13% (Siegel et al., 2015).

For advanced BTCs, chemotherapy is the main systemic therapy. Early studies found fluoropyrimidine, platinum, and gemcitabine to be effective drugs for the treatment of advanced BTCs. Subsequently, based on the ABC-02 clinical trial, the combination of gemcitabine plus cisplatin (CDDP-GEM) became the recognized reference regimen for first-line treatment of patients with advanced BTCs (Valle et al., 2010). Numerous studies have been conducted over the last 10 years, and this combination remains the standard of care worldwide (Banales et al., 2020; Rizzo and Brandi, 2021a; Rizzo et al., 2021). Although cisplatin appears to be more effective, it is more toxic (Fiteni et al., 2014), and the gemcitabine and oxaliplatin (GEMOX) regimen has been widely used as first-line treatment for patients unsuitable for cisplatin.

In recent times, an open-label, randomized, phase 3, non-inferiority trial investigated the clinical effectiveness and safety of XELOX (capecitabine plus oxaliplatin) versus GEMOX therapy as first-line therapy for advanced BTCs (Kim et al., 2019). The results indicated that the median overall survival (OS) time was 10.6 months (95% confidence interval [CI], 7.3–15.5) in the XELOX group and 10.4 months (95% CI, 8.0–12.6) in the GEMOX group, with no statistically significant difference in OS curves between the two groups (*p* = 0.131). Meanwhile, the frequency of hospitalization was significantly lower in the XELOX group than in the GEMOX group (*p* < 0.001). Based on these results, XELOX therapy was approved for the first-line treatment of BTC.

Despite the survival benefits of available treatments for BTCs, the financial impact remains considerable. At present, the cost-effectiveness of XELOX versus GEMOX as first-line solutions for BTC has not been evaluated. To improve the effective use of limited healthcare resources and to help evidence-based healthcare decisions, we conducted a health economics evaluation of disease-related therapies.

Significant geographic differences in the incidence of BTCs have been reported, with higher prevalence in Thailand, Japan, China, and South Korea compared to Western countries (Rizzo and Brandi, 2021b). It is evident that financial expenditures for disease treatment will increase the burden on the medical insurance system of various countries. Thus, using the results of Kim’s trial (Kim et al., 2019), this study assessed the cost-effectiveness of XELOX therapy versus GEMOX therapy in the treatment of BTC from the perspective of Chinese healthcare payers.

## Methods

### Model Building

**FIGURE 1:**
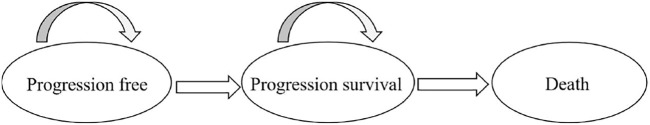
The Markov state transition model. At the beginning of each Markov cycle, all patients entered the model in the progression-free survival (PFS) with a stable disease state and immediately commenced treatment. From this state, patients could either remain in a PFS state or experience progression and enter progression survival (PS). Patients in the PS could either remain in a PS state or transition to death. PS indicates the progression of the disease.

A decision-analytic model was developed to compare the costs and effectiveness of XELOX with those of GEMOX for advanced BTCs. As reported in the trial by Kim et al. (2019), treatment was repeated every 3 weeks for both groups for a total of eight cycles and was discontinued in cases of disease progression. The following two first-line treatment options were evaluated using the model: 1) XELOX: 1,000 mg/m^2^ capecitabine was administered orally twice daily (bid) on days 1–14, and 130 mg/m^2^ oxaliplatin as a 120-min infusion on day 1; and 2) GEMOX: 1,000 mg/m^2^ gemcitabine was administered as a 100-min infusion on days 1 and 8, and 100 mg/m^2^ oxaliplatin as a 120-min infusion on day 1. The analysis was conducted from the perspective of the Chinese healthcare system, with a lifetime horizon. We considered only direct medical costs. Each model cycle represented 21 days. The costs and effectiveness outcomes were discounted at 5% annually. A three-health-state Markov model was developed as follows: progression-free survival (PFS) with responsive/stable disease, progression survival (PS), and death (Figure 1) (Tsukiyama et al., 2017). Patients were in PFS at the initial stage of the model, and each cycle was left in PFS or converted to PS according to the transfer probability. Entering PS can only be in PS or into a state of death. After progress, the patients entered the best support treatment. Parametric survival curve fitting was performed in R (version 4.1.1) software, and the Markov model was developed and run in TreeAge Pro 2020.

### Effectiveness Parameters and Utility Estimates

The three health states of the transfer probabilities of BTCs were estimated based on the OS and PFS Kaplan–Meier (KM) curves of Kim’s trial (Kim et al., 2019). The GetData Graph Digitizer software package was used to reconstruct the individual data, and the RStudio software was used to perform statistical analyses. The Weibull distribution was chosen to extrapolate PFS and OS. The survival functions were used to calculate the transfer probabilities among the states. These parameters were substituted into the equation P(t) = 1 − exp[λ(t − 1)^γ^ − λt^γ^] to calculate the transition probability. Weibull distribution scale parameter λ and shape parameter γ are shown in Table 1.

**TABLE 1:** Key model parameters.

	Shape	Scale	Distribution	Source
PFS				
XELOX	0.201	0.690	Weibull	9
GEMOX	0.199	0.739	Weibull	9
OS				
XELOX	0.068	0.958	Weibull	9
GEMOX	0.038	1.260	Weibull	9

Abbreviations: PFS, progression-free survival; OS, overall survival

Quality-adjusted life-year (QALY) was identified as the primary health outcome. It is often referred to as utility (the health-state utility ranges from 0 [death] to 1 [complete health]) (Li et al., 2021). Since BTC is a relatively rare cancer, no independent health status utility values have been published in this area. We obtained the values of the health utility values from the previously published literature (Roth and Carlson, 2012; Tsukiyama et al., 2017) (Table 2). A discount rate of 5% was applied to the QALY calculations.

**TABLE 2:** Ranges and distribution of other parameters.

Variable		Baseline value	Range	Dis	References
Utilities	PFS	0.69	0.455–0.925	Beta	10,12
	PS	0.71	0.455–0.965	Beta	10,12
SAEs rates					
XELOX therapy	Stomatitis	0.01	0.008–0.012	Beta	9
	Hand-foot syndrome	0.03	0.024–0.036	Beta	9
	Neutropenia	0.04	0.032–0.048	Beta	9
	Thrombocytopenia	0.09	0.072–0.108	Beta	9
	Asthenia	0.02	0.016–0.024	Beta	9
	Anorexia	0.02	0.016–0.024	Beta	9
GEMOX therapy	Nausea	0.01	0.008–0.0012	Beta	9
	Vomiting	0.01	0.008–0.0012	Beta	9
	Diarrhea	0.01	0.008–0.0012	Beta	9
	Stomatitis	0.01	0.008–0.0012	Beta	9
	Neutropenia	0.14	0.112–0.0168	Beta	9
	Neutropenic fever	0.01	0.008–0.0012	Beta	9
	Thrombocytopenia	0.11	0.088–0.0132	Beta	9
	Elevated AST/ALT	0.02	0.016–0.0024	Beta	9

Abbreviations: PFS, progression-free survival; PS, progression survival; SAEs, serious adverse events, Dis, Distribution; AST, aspartate transaminase; ALT, alanine transaminase.

### Cost Estimates

The costs involved in this study mainly included direct medical expenses, such as drug expenses, follow-up testing, management of treatment-related serious adverse events (SAEs), best supportive care (BSC), and terminal care (Table 3). The Chinese yuan (CNY) was converted to the US dollar using an average exchange rate in 2020 of 6.8976 CNY = 1.00 US dollar (National Bureau of Statistics of China, 2021).

**TABLE 3:** Cost parameters.

Input Parameter		Value ($)	Range ($)	Dis	Source [ref.]
Drug cost of XELOX	Capecitabine	3.19	2.55–3.83	Gamma	20
	($/500 mg)				
	Oxaliplatin	304.45	243.56–365.34	Gamma	20
	($/50 mg)				
Drug cost of GEMOX	Gemcitabine ($/1.0g)	229.94	183.95–275.93	Gamma	20
	Oxaliplatin ($/50 mg)	304.45	243.56–365.34	Gamma	20
Drug administration	XELOX	5.8	4.64–6.96	Gamma	#
	GEMOX				
SAEs costs/unit	Nausea	66.34	53.07–79.61	Gamma	21
	Vomiting	66.34	53.07–79.61	Gamma	21
	Diarrhea	13.27	10.61–15.92	Gamma	21
	Stomatitis	-	-	Gamma	-
	Hand-foot syndrome	4.08	3.27–4.9	Gamma	21
	Neutropenia	3974.49	3179.59–4769.39	Gamma	22
	Neutropenic fever	2231.69	1785.35–2678.03	Gamma	22
	Thrombocytopenia	6526.06	5220.85–7831.27	Gamma	22
	Elevated AST/ALT	60.22	48.17–72.26	Gamma	21
	Asthenia	3.06	2.45–3.67	Gamma	21
	Anorexia	26.54	21.23–31.84	Gamma	21
Follow-up tests costs/cycle	Hospitalization cost	14.5	11.6–17.4	Gamma	#
	Laboratory tests	30.45	24.36–36.53	Gamma	#
	CT scan	65.24	52.19–78.29	Gamma	#
BSC cost		123.69	98.95–148.42	Gamma	23
Terminal care cost		1,567.89	1,254.31–1881.47	Gamma	23

Abbreviations: XELOX, capecitabine plus oxaliplatin; GEMOX, gemcitabine plus oxaliplatin; SAEs, serious adverse events, Dis, Distribution; AST, aspartate transaminase; ALT, alanine transaminase; CT, computerized tomography; BSC, best supportive care. #: Hospital charges

We used a mean body surface area (BSA) of 1.72 m^2^ (Liubao et al., 2009) to calculate the doses of gemcitabine (1,000 mg/m^2^ BSA), oxaliplatin (100 mg/m^2^ BSA or 130 mg/m^2^ BSA), and capecitabine (1,000 mg/m^2^), which was based on the trial by Kim et al. (2019). The prices of drugs in China were obtained from the Yaozhi network (Yaozh, 2021). Routine disease management costs, including biochemical tests, blood routine examinations, and computerized tomography (CT), are calculated according to the actual charging standards of local medical institutions. This study considered the cost of follow-up testing for the PFS state and calculated it throughout the treatment process. The costs involved in this study use US dollars as the unit.

Adverse event (AE) costs were taken from the previously published studies. Grade 3/4 AEs were defined as SAEs. Only SAEs were considered in our study (Kim et al., 2019), which included nausea, vomiting, diarrhea, stomatitis, hand–foot syndrome, neutropenia, neutropenic fever, thrombocytopenia, elevated aspartate transaminase (AST)/ alanine transaminase (ALT), asthenia, and anorexia. These AE costs were calculated by multiplying the estimated incidence rate of each AE by the corresponding unit treatment cost (Wu et al., 2014; Qin et al., 2018; Zheng et al., 2018). All AEs were assumed to occur in the first cycle of treatment. The incidence rates of each AE are listed in Table 2, and all unit AE costs used in the base analysis are listed in Table 3.

Because of the lack of recommended second-line treatment options, the costs of treatment after the disease progresses consist of BSC and terminal care. We assume that terminal care costs were considered as a one-time cost in the final state. All costs were inflation-adjusted to 2020 US dollars depending on the Chinese Consumer Price Index healthcare services group.

### Sensitivity Analysis

The uncertainty of the model was tested using univariate sensitivity analysis and probabilistic sensitivity analysis (PSA). Univariate sensitivity analysis was carried out to evaluate the influence of each applicable parameter on the model. The parameter was adjusted successively to its respective low and high values, which were obtained from the CIs or 20% variance of the hypothetical baseline case value. We used a 1,000-time Monte Carlo simulation to perform PSA, with variables simultaneously varied, with a specific pattern of distribution. The ranges and distribution of the parameters used in the sensitivity analyses are shown in Tables 2 and 3, respectively.

Owing to the lack of an acceptable threshold for the Chinese population, according to the World Health Organization’s recommendations for cost-effectiveness analysis, this study will take 3 × China’s gross domestic product (GDP) per capita in 2020, which is US $31,315.24, as the threshold value.

## Results

### Base Case Results

The results of the basic analysis in Table 4 show that the QALYs for the XELOX and GEMOX groups were 0.66 and 0.54, respectively. The total cost of XELOX treatment was US $12,275.51 in China, which was lower and more effective than the GEMOX regimen, thereby making it the preferred regimen.

**TABLE 4:** Base case results in China.

Result	XELOX	GEMOX	Incremental
QALY	0.66	0.54	0.12
The total cost of the regimen $	12275.51	13649.62	−1,374.11
ICER, US$/QALY	−12070.42		

Abbreviations: XELOX, capecitabine plus oxaliplatin; GEMOX, gemcitabine plus oxaliplatin; QALYs, quality-adjusted life-years; ICER, incremental cost-effectiveness ratios.

### Sensitivity Analyses

**FIGURE 2:**
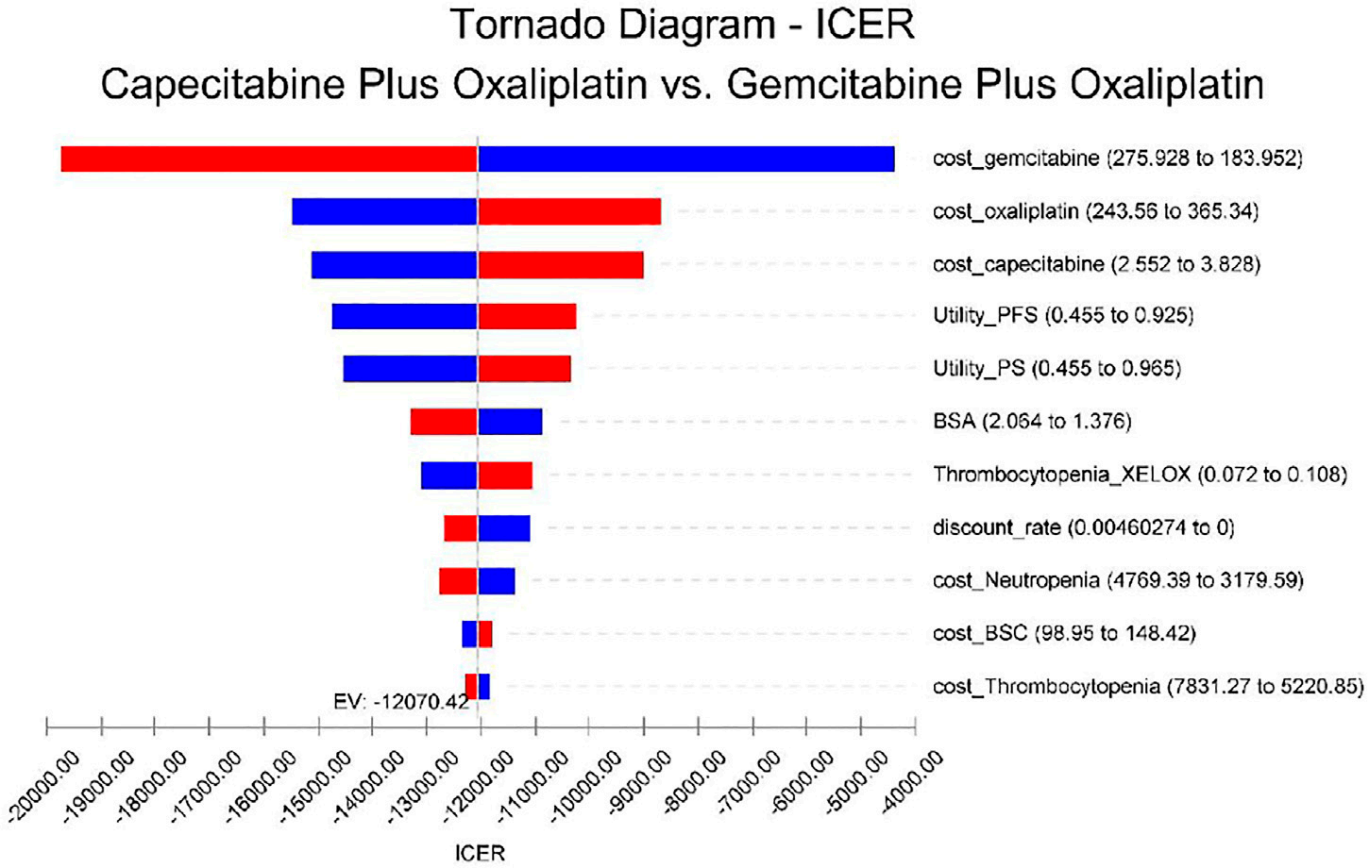
Univariate sensitivity analysis. The tornado plots show the ICER of the XELOX therapy versus the GEMOX therapy for different input parameters in the model. The horizontal axis of the figure represents the range of influence of each element on the results, and the vertical axis shows the name of each uncertainty factor. The horizontal bars indicate the value of the effect of each element on the result and the value of the effect of each element itself. The effects of the factors on the ICER are listed in descending order of significance. Abbreviations: EV, expected value; BSA, body surface area; BSC, best supportive care; PFS, progression-free survival; and PS, progression survival.

Tornado plots are used to show the results of univariate sensitivity analyses to determine the parameters of the model, which have the greatest impact on incremental QALY and cost. The cost of gemcitabine has the greatest impact on the incremental cost-effectiveness ratio (ICER) results, and the remaining sensitive parameters are, in order, the cost of oxaliplatin, the cost of capecitabine, and the utility values of patients in PFS versus PS status, with the other parameters having little impact, as shown in Figure 2. In short, changing the parameter values within a certain range has a limited effect on the outcome.

**FIGURE 3:**
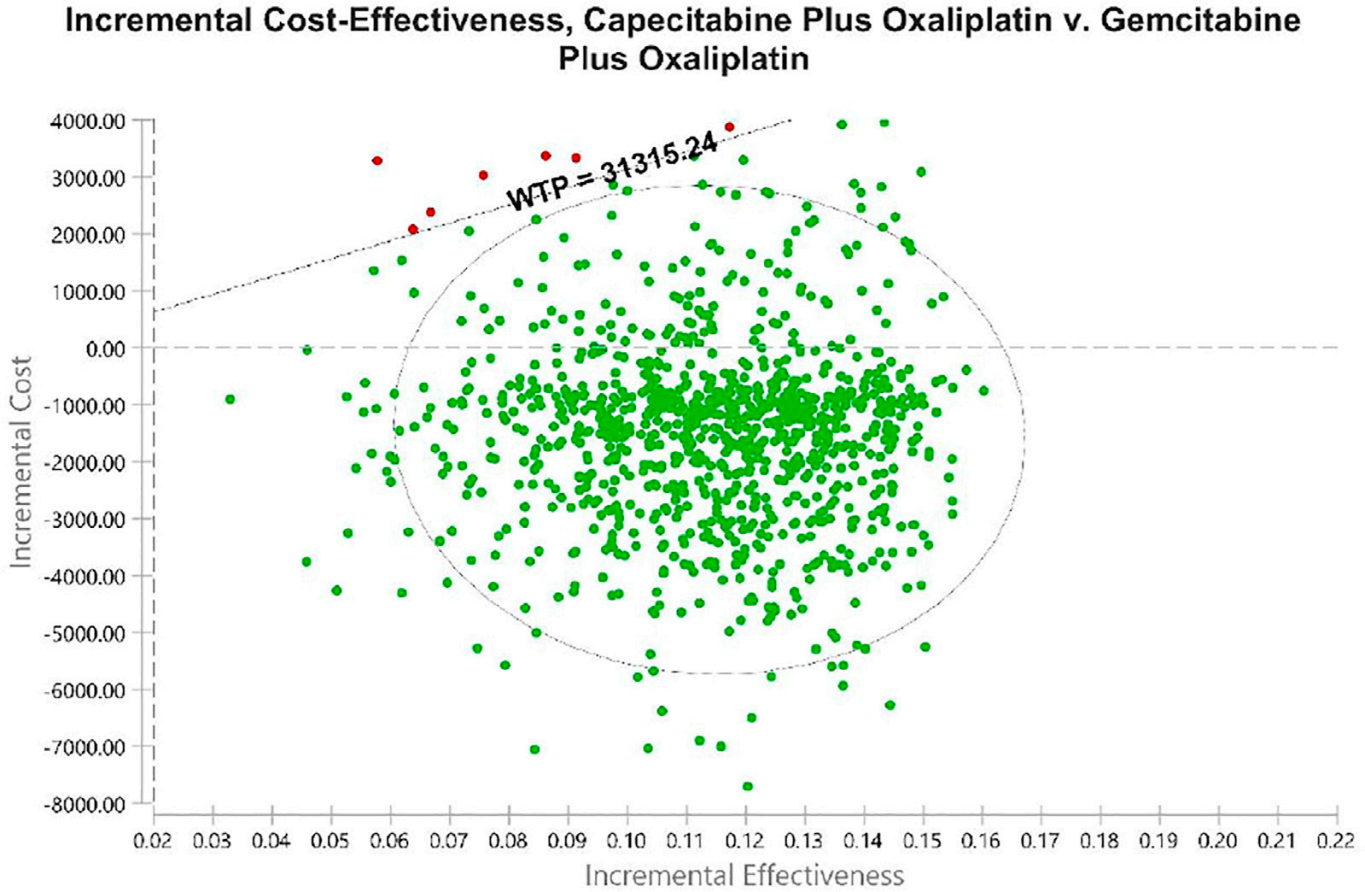
Probabilistic sensitivity analyses. Dots indicate the results of Monte Carlo simulations, and ellipses indicate 95% confidence intervals. The diagonal line shows WTP. Dots located below the diagonal line indicate cost-effectiveness for the experimental group compared to the corresponding control group. Abbreviations: WTP, willingness to pay. All costs are in United States dollars.

**FIGURE 4:**
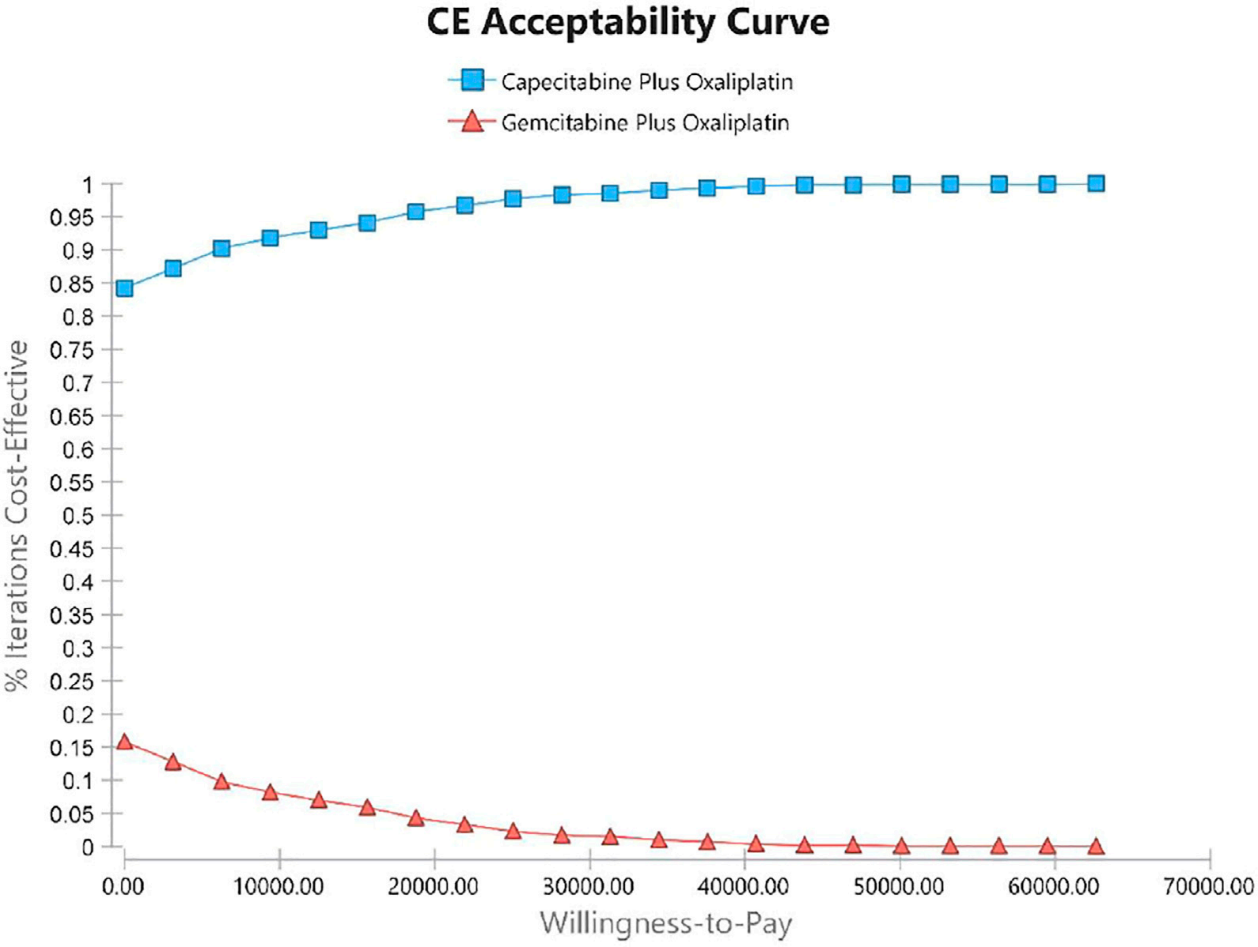
Cost-effectiveness acceptability curves (CEACs). The CEAC represents the economic probability of a drug. The curve shows the percentage of cost-effectiveness simulated using different treatment solutions.

Using PSA, the effect of all model input parameters on the results of the study was observed, which were constantly changing and met different distributions. According to the PSA results, XELOX treatment is extremely cost effective compared with GEMOX treatment in the first line of treatment. Figure 3 shows scatterplots with a sloping line as the willingness-to-pay (WTP) threshold line. The 95% CI for ICER is represented by an ellipse. In China, when the WTP threshold is adjusted to 1–3 × GDP per QALY, the probability of the cost-effectiveness of XELOX treatment is 92.1, 96.2, and 99.8%, respectively. Based on the cost-effectiveness acceptable curve, XELOX treatment is a superior choice in China (Figure 4).

## Discussion

Advanced BTC is a rare disease characterized by a high rate of recurrence and distant metastasis. At present, there are few cost-effectiveness evaluations of first-line treatment options for advanced BTCs. The high prices of anti-cancer drugs have led to a sharp increase in the consumption of medical resources, which has troubled clinicians and medical managers. Healthcare spending in high-income countries, such as the United States and Europe, has been increasing year by year (Laviana et al., 2020; Godman et al., 2021). They have explored potential approaches to pricing cancer therapeutics in the hope of maintaining a sustainable impact on the healthcare system (Godman et al., 2021). Low- and middle-income countries (LMICs) are also actively addressing the rising costs of cancer treatment. In particular, as cancer treatments diversify, the selection of novel antineoplastic drugs, such as trastuzumab, may not be cost effective in LMICs (Gershon et al., 2019; Al-Ziftawi et al., 2021). Therefore, it is very important to evaluate treatment options economically and to use limited medical resources rationally and effectively.

To date, the CDDP-GEM regimen remains the standard of care for the first-line treatment of advanced BTC worldwide. In actual clinical practice, the more common hematologic and renal toxicity of cisplatin leads to treatment discontinuation (Yamada et al., 2015; Markussen et al., 2020), so clinicians tend to choose oxaliplatin for frail patients. According to Fiteni’s (Fiteni et al., 2014) study, oxaliplatin-based regimens are relatively uniform (oxaliplatin doses range from 80 to 100 mg/m^2^), while CDDP-GEM regimens have significant heterogeneity, with cisplatin doses ranging from low (25–35 mg/m^2^) to high (60–80 mg/m^2^). Multiple doses of cisplatin would interfere with our study. Compared to the CDDP-GEM regimen, which has comparable efficacy but an increased incidence of adverse effects, an oxaliplatin-based combination regimen is a better choice.

Our results showed that XELOX treatment is a better option in China because it is less costly and more effective. One-way sensitivity analysis showed that drug price was the parameter that caused the greatest change in ICER values. The costs of capecitabine and gemcitabine were the main influencing factors. This was consistent with the findings of several published studies. For instance, in the study by Atieno et al. the drug was the main factor contributing to the cost of cancer treatment (Atieno et al., 2018). However, in our study, the relationship between ICER and WTP thresholds remained unchanged by varying the values of key parameters within reasonable limits. As far as we know, medical insurance in China has covered as many people as possible. However, there were great differences in reimbursement rates between different regions and different types of medical insurance. Choosing a treatment option with better results and lower costs is conducive to the rational use of health insurance. Therefore, we suggest that health insurance authorities should appropriately increase the reimbursement rate of the XELOX regimen for BTC patients, which will help save health insurance costs and benefit more BTC patients.

To date, two cost-effectiveness analyses of BTC have been published, but they provided comparisons with the CDDP-GEM regimen and gemcitabine monotherapy strategy. Roth’s study (Roth and Carlson, 2012) evaluated the cost-effectiveness of adding cisplatin to standard gemcitabine therapy from the US societal perspective and revealed that, relative to gemcitabine monotherapy, the ICER for the combination was US $59,480 per QALY, which is cost effective. However, combination therapy is less costly than monotherapy for advanced BTC in Japan (Tsukiyama et al., 2017). The reason may be that the cost of long-term palliative care in Japan is higher than that in the United States. In contrast to these two studies, our study compared the economics of XELOX and GEMOX in BTC treatment. Overall, from the perspective of the Chinese payers, XELOX therapy was a cost-effective strategy for the first-line systemic treatment for BTC.

Our study has some limitations. First, clinical data were obtained from a phase 3 trial conducted in Korea (Kim et al., 2019), which was limited to Asian patients. In previous studies (Valle et al., 2010; Lee et al., 2012), the combination of gemcitabine and platinum analogs showed antitumor activity in both Asian and non-Asian populations. These factors may have had a slight influence on our results. Second, our literature search did not identify any studies reporting the practical value of health status in patients with advanced BTC. Therefore, we used data from Roth’s study (Roth and Carlson, 2012) on hepatocellular carcinoma, and the authors suggest that similar health-state utility values exist for patients with advanced BTC. We referred to previous literature (Roth and Carlson, 2012; Tsukiyama et al., 2017) to vary health utility values over a considerable range (±0.34) to account for the uncertainty of this factor. One-way sensitivity analysis showed that changes in health utility values had only a slightly significant effect on the model. Third, our study may not be timely enough. The results of the TOPAZ-1 (NCT03875235) trial have just been published, with an objective response rate (ORR) of 73.4% in the duvalizumab plus chemotherapy (D + CDDP-GEM) arm and a median OS of 18.1 months (Oh et al., 2022), which is significantly better than the historical data for the CDDP-GEM regimen (11.7 months). However, the final analysis has not yet been published, which hindered our study. We will follow up further in the future to ensure the timeliness of the study. Fourth, to reduce the impact of parameter uncertainty, we simplified the model and made several assumptions. There may be different treatment options after disease progression, but currently, there is no uniform recommended treatment option after first-line treatment for advanced BTC. Many studies are exploring the feasibility of alternative endpoints with survival (Prasad et al., 2015; Wild et al., 2016; Paoletti et al., 2020); however, the results are unfortunate, and there is limited evidence for using surrogate endpoints (e.g., PFS) as a proxy for overall tumor survival. Therefore, we did not consider drug options after disease progression. Our study also did not discuss the decrease in utility values caused by adverse effects. However, sensitivity analyses showed that changes in utility values did not qualitatively alter outcomes. Therefore, the state from PFS to death was not considered in the model. Fifth, indirect costs, such as loss of income from discontinuation and premature death, were not considered in our analysis because the high variability of the condition makes it difficult to calculate accurately. Drug prices and treatment costs were obtained from the previously published literature or local data, which may not apply to all regions. To avoid the impact of these costs on the model, we varied treatment costs over a considerable range (±20%) in the one-way sensitivity analysis. The variation was mediated by these factors and was limited. Sixth, modeling long-term OS in patients with advanced BTC using the Weibull distribution is an unavoidable limitation of our study. In the future, with a more appropriate approach, we could better fit the long-term survival data of patients.

Although there were some limitations to our research, the variables in the model did not affect the final result. Sensitivity analysis showed that the probabilities, utilities, and costs in the model had a limited impact on the final results, which illustrates the robustness of the model.

## Conclusion

The XELOX regimen is more cost effective as a first-line treatment for advanced BTC than the GEMOX regimen from the perspective of the Chinese health service systems. However, for specific patients, clinical decision-makers need to consider all effective treatment options for advanced BTC.

## Data Availability

The original contributions presented in the study are included in the article/supplementary material further inquiries can be directed to the corresponding authors.

## Abbreviations

(AE), adverse event; (ALT), alanine transaminase; (AST), aspartate transaminase; (BSA), body surface area; (BSC), best supportive care; (BTC), biliary tract cancer; (CDDP-GEM), gemcitabine plus cisplatin; (CNY), Chinese yuan; (CT), computerized tomography; (XELOX), capecitabine plus oxaliplatin; (GEMOX), gemcitabine plus oxaliplatin; (ICER), incremental cost-effectiveness ratio; (LMICs), Low- and middle-income countries; (OS), overall survival; (ORR), objective response rate; (PFS), progression-free survival; (PS), progression survival; (PSA), probabilistic sensitivity analysis; (QALY), Quality-adjusted life-year; and (SAE), serious adverse event.
